# Psychometric Modeling to Identify Examinees’ Strategy Differences during Testing

**DOI:** 10.3390/jintelligence12040040

**Published:** 2024-03-29

**Authors:** Clifford E. Hauenstein, Susan E. Embretson, Eunbee Kim

**Affiliations:** 1 Department of Neurology, Division of Cognitive Neurology/Neuropsychology, The Johns Hopkins University School of Medicine, Baltimore, MD 21218, USA; chauens2@jh.edu; 2 Department of Psychology, Georgia Institute of Technology, Atlanta, GA 30332, USA; eunbee.kim@gatech.edu

**Keywords:** test-taking strategies, psychometric modeling, score interpretability

## Abstract

Aptitude test scores are typically interpreted similarly for examinees with the same overall score. However, research has found evidence of examinee differences in strategies, as well as in the continued application of appropriate procedures during testing. Such differences can impact the correlates of test scores, making similar interpretations for equivalent scores questionable. This study presents some item response theory (IRT) models that are relevant to identifying examinee differences in strategies and understanding of test-taking procedures. First, mixture IRT models that identify latent classes of examinees with different patterns of item responses are considered; these models have long been available but unfortunately are not routinely applied. Strategy differences between the classes can then be studied separately by modeling the response patterns with cognitive complexity variables within each class. Secondly, novel psychometric approaches that leverage response time information (in particular, response time residuals) in order to identify both inter and intraindividual variability in response processes are considered. In doing so, a general method for evaluating threats to validity is proposed. The utility of the approach, in terms of providing more interpretable performance estimates and improving the administration of psychological measurement instruments, is then demonstrated with an empirical example.

## 1. Introduction

Response process heterogeneity can represent a critical threat to one or more aspects of the validity of performance claims made from psychological measurement instruments (see Eignor 2014). While sources of item cognitive complexity may vary systematically at different difficulty levels, the majority of scoring protocols for psychological measures assume that *equal* scores imply the same response processes. However, evidence suggests that it is not uncommon for examinees to adopt a range of different strategies and approaches. 

Apart from concerns regarding fundamentally different solution strategies (Bethell-Fox and Shepard 1988; Embretson 2007; Gluck and Fitting 2003; Mislevy and Verhelst 1990; Schultz 1991), response processes also may be influenced by other construct-irrelevant factors like fatigue (Kingston and Dorans 1982; Davis and Ferdous 2005; Albano 2013), attentional lapses (e.g., Smallwood et al. 2003, 2004; Unsworth et al. 2021), shifts in speed–accuracy tradeoff, or initial unfamiliarity with task demands (e.g., a ‘warmup effect’). When these processes are not adequately addressed either by modifying administration procedures (see Goldhammer 2015) or applying flexible measurement models, the estimates of ability will be obfuscated by these non-optimal response processes, mitigating their utility towards predicting performance in other contexts. 

Thus, both between- and within-person differences in solution strategies can affect performance levels and produce ability estimates whose interpretive value is compromised. Fortunately, as noted below, there are models available to identify these differences.

### 1.1. Between-Person Differences in Strategies

Background. Although much work remains to implement methods to mitigate these threats to validity in practice, the psychometric literature has provided some modeling techniques to account for these issues. For example, mixture distribution models (Bolt et al. 2002; Rost and von Davier 1995; von Davier 2009) based on item response theory (IRT) have been developed to identify latent classes of examinees who differ in item response strategies. Mixture models have also been extended to continuous variables (Zopluglu 2020). These models are based on the fit of individual examinee responses to the IRT model used to estimate trait level and item difficulty. Examinees with responses that do not fit will be part of additional latent classes with different patterns of item difficulty. 

Several studies have found that the latent classes obtained from mixture models differ not only in cognitive processes, as determined by the relationships of item difficulty with various item cognitive complexity features and response time, but also in the correlations of trait level with external variables. For example, Embretson (2023) found four classes with varying patterns of item difficulty on a test of spatial ability. Modeling the item difficulty patterns from item cognitive complexity variables revealed differences in spatial task processing strategies between classes, which was further supported by the varying external correlations of trait levels with other types of aptitude tests. Thus, the results suggested that individuals in only one class used a primarily spatial visualization strategy. The other classes apparently adopted either verbal solution strategies or guessing behavior. Thus, the practical meaning of performance estimates (i.e., validity) may be specific to each class.

Goals for Study 1. In Study 1, the impact of multiple latent classes identified using mixture models on the aspects of validity is examined for a non-verbal intelligence test. A mixture model that identifies patterns of item difficulty is applied to analytic reasoning consisting of matrix items. Both person and item correlations are examined in the separate latent classes to indicate the nature of strategy differences.

### 1.2. Within-Person Changes in Strategies

Of course, these mixture distribution models do not specifically allow for intra-individual variability in response processes during testing. One of the earliest efforts to free this constraint was proposed by Yamamoto and Everson (1997); they proposed a *change-point* model that attempted to identify at what time point a respondent shifted from a construct-driven response style to a disengaged, guessing approach. This represented a time series extension of the earlier HYBRID model (Yamamoto 1989). Fundamental to Yamamoto’s approach is the notion that engaged, construct-driven response behavior should be captured by a traditional measurement model (i.e., producing variance in both item difficulties and person ability estimates), while disengaged responding would show poor fit to such a measurement model and be classified into a distinct latent class. The change-point approach of Yamamoto and Everson still carries influence; Goegebeur et al. (2008) offered a similar model to identify gradual shifts in response style. However, these approaches were limited in that they did not allow for multiple change points or regressions to previous states.

Response time analysis. More recently in the psychometric literature, response time information has been considered as an additional source of information to assist in the identification of disengaged responding, assuming its direct relation to speed-accuracy tradeoff, fatigue, attentional lapses, the warmup effect, and guessing. Furthermore, the inclusion of the richer, continuous response time data allows for more complex transition patterns to be evaluated and also improves the precision with which examinee response styles can be identified.

Part of the recent emphasis on response time is borne out of a move to digital administration of tests, allowing for the easy collection of precise response time information. Another part of the emphasis has also been due to the influential response time model of van der Linden (2006), which established a framework for deriving both person-level speed factors and item-level time intensity factors. The formulation by van der Linden can be considered a response time analog of a traditional measurement model, in that it established an additive relation between person- and item-level effects to produce observed response times. Generalizations of the van der Linden model have included both inter and intra-examinee variance in response processes. For example, Zhu et al. (2023) integrated a multiple change-point model within van der Linden’s model to identify abrupt shifts in the person speed factor during testing. The approach was motivated by the notion that each examinee might shift from a slow process at the onset of testing (while in the midst of identifying an appropriate strategy), to a moderately paced process during the middle of a test, to a rapid (guessing) process during the latter stages of a test. Molenaar et al. (2016) similarly used van der Linden’s parameterization to identify different response time states, and then modeled the transition between these states according to a Markov process.

As a somewhat alternative approach, van der Linden and Guo (2008) proposed adopting the van der Linden model but shifting focus towards the *residuals* of the model as indices of the response process. From this perspective, the distinction is between engaged versus erratic response processes, with larger residual terms (in absolute value) implying a more erratic response process. Such an approach provides a more general method of identifying responses potentially tarnished by construct-irrelevant processes since it simultaneously identifies both uncharacteristically rapid responses and uncharacteristically protracted responses. Thus, rapid guessing behavior, warm-up processes, attentional lapses, and fatigue-contaminated responses are all simultaneously classified into a singular, erratic response state.

Goals for Study 2. The generalized approaches to the van der Linden model, as described above, have not been formally considered from an applied perspective that addresses two important questions:

(1) Can the transition patterns be directly modeled and hence used to inform future test administration practice?
(2) Can information from these transition patterns be used to adjust the ability estimates in an effort to improve the underlying validity of the measure?

To our knowledge, previous work has not evaluated the utility of a residual-based approach in direct relation to improvements in a validity coefficient (i.e., improving external relationships with performance on theoretically related measures).

In Study 2, a modeling protocol that jointly considers accuracy and response time data is proposed, with an emphasis on response time residuals, to address the two questions posed above. Study 2 intends to extend Study 1 by offering a more refined, diagnostic evaluation of validity at the person level by directly leveraging different response time patterns. The impact of this approach on the external validity of the aptitude test as a whole is considered, and differences from mixture psychometric approaches (e.g., Study 1 methods) are discussed.

## 2. Study 1: Mixture Models to Identify Strategy Differences

### 2.1. Background: Mixture Models

As noted above, mixture models are used in item response theory (IRT) to identify individual differences in item response strategies. Mixture Rasch models, e.g., (Rost and von Davier 1995) identify latent classes of examinees based on fit statistics using the Rasch model to predict item responses. For example, the Rasch model gives the probability that person *j* solves item *i* as follows:

(1) $$p(X_{ij}=1|\theta_{j},\beta_{i})=\frac{\exp\left(\theta_{j}-\beta_{i}\right)}{1+\exp\left(\theta_{j}-\beta_{i}\right)}$$

where *θ_j_* = trait level of person *j* and *β_i_* = difficulty of item *i*.

Fit statistics in mixture model software, such as winMIRA2001 (von Davier 2001) and mdltm (von Davier 2005), identify misfitting persons to form new latent classes. Misfit is obtained when a person’s response accuracies are not well predicted from their trait estimate and item difficulties within a class. Thus, the misfitting person has a different pattern of item difficulty than others in the same class. Hence, misfitting persons form a new latent class, and item difficulty is computed separately for that new class. The overall fit of the data to the multiclass model is then evaluated and a new class is determined for misfitting persons.

The mixture IRT model for binary data is presented as follows:

(2) $$p\left(X_{ij}=1|\theta_{j_{g}},\beta_{i_{g}}\right)=\sum_{g}\pi_{g}\frac{\exp\left(\theta_{j_{g}}-\beta_{i_{g}}\right)}{1+\exp\left(\theta_{j_{g}}-\beta_{i_{g}}\right)}$$

where $\theta_{j_{g}}$ represents the trait level of person *j* in group g, $\beta_{i_{g}}$ represents the difficulty of item *i* in group g, and $\pi_{g}$ reflects the probability for group g. Notice that the group subscript for item difficulty supports different patterns of values between groups.

In Study 1, the mixture model specified in Equation (2) is used to identify potential strategy differences between persons taking the Abstract Reasoning Test (ART), a test of analytic reasoning. Compared to the spatial test used by Embretson (2023) to examine varying cognitive strategies, ART measures a more general aspect of intelligence (i.e., non-verbal reasoning). The number of classes needed to provide adequate fit is assessed by evaluating the improvements in fit by adding successive new classes. The winMIRA software is used to identify the classes, as the authors have found it to produce interpretable class differences (e.g., Embretson 2023). But it should be noted that many other software packages are available (e.g., psychomix; Frick et al. 2012; Frick 2022). Additional analysis of individual differences between classes and their correlates, as well as the relationships of item difficulty to other variables, is examined to determine the nature of the classes. That is, after latent classes with different patterns of item difficulty are identified, mathematical modeling will be used to examine potential strategy differences. As indicated by Busemeyer and Diederich (2010), mathematical modeling of responses and response times has been the most prevalent method in cognitive psychology to study strategies.

### 2.2. Method

**Figure 1:**
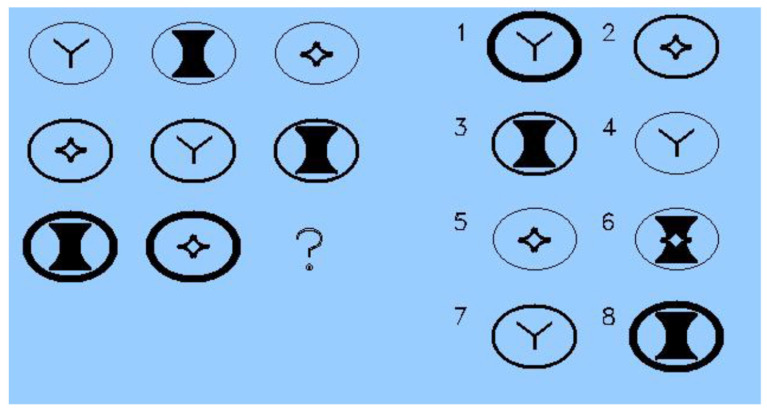
Example of ART item.

**Test.** ART was used to examine strategy differences among examinees. ART consists of 3 × 3 matrices with a missing element for which inclusion depends on finding the relationships between the elements. ART includes eight response options for the missing element. Five different types of relationships and/or their combination may be involved. The relationships, ordered for cognitive complexity, are (1) constant in a row, (2) pairwise progressions, (3) figure addition/subtraction, (4) distribution of three, and (5) distribution of two. The ART item in Figure 1 involves two relationships (the answer is #1); that is, constant in a row (i.e., circle outline thickness) and distribution of three (each of three interior objects appear once in each row and column). Other items may involve just one or as many as three separate relationships of varying types between the elements in the matrix. The version of ART used in the current study consists of 30 items.

**Test administration.** ART was administered to a group of young adults in a laboratory with multiple computers and monitored by a test administrator. The instructions provided examples of the various relationships. Both item responses and response time were available.

**Examinees.** The examinees were 301 military recruits who took ART and other tests on a voluntary basis. Background variables were available, including the Armed Forces Qualification Test (AFQT) score. AFQT is a measure of general intelligence that has high stakes for examinees; that is, it determines enlistment eligibility for the military. Also, the external relationships aspect of validity is well established for the AFQT scores.

**Estimation.** The winMIRA software was used to identify the classes. In winMIRA, item parameters for the Rasch model within each latent class were estimated by conditional maximum likelihood with the item mean set to zero. The overall fit indices include the log-likelihood of the data, chi square tests between successive numbers of classes, and an AIC index. Individual examinee fit indices are also given. Finally, because the natural floor effect of response time data generally results in a positively skewed distribution, a log transform (lnRT) is applied to the response time data to produce more normally distributed values.

### 2.3. Results

Table 1 presents the statistics on mixture models with varying numbers of classes. The chi square test shows significant improvement in fit by estimating two classes versus one class, and by estimating three classes versus two classes. The three-class solution was significant at the .05 level. However, the smallest class size (.23) had 70 examinees. Due to the decreasing significance levels and class sizes, a four-class solution was not attempted. Thus, the three-class solution was retained for further analysis.

Table 2 presents descriptive statistics and correlations of the examinee trait estimates in the three classes. The classes differ significantly (*F*(2, 298) = 142.552, *p* < .001) in mean trait levels. Class 1, the largest class, has a trait level ($\bar{\theta })$ slightly below the mean item difficulty of zero, which yields a mean item-solving probability of .442 (obtained by applying the Rasch model formula in Equation (1) to responses in this class).

Class 2, on the other hand, has a much higher mean trait level, which corresponds to a mean item-solving probability of .844. The trait level for Class 3 falls between the other two classes, with a trait level that corresponds to a mean item-solving probability of .678.

The classes also differed significantly in mean lnRT per item (*F*(2, 298) = 21.131, *p* < .001), with Class 1 having the lowest value (equivalent to an RT of 20.860 s) and Class 2 having the highest value (equivalent to an RT of 31.375 s). Class 3 spent the equivalent of 25.790 s per item. Finally, the classes also differed significantly in AFQT scores (*F*(2, 298) = 27.912, *p* < .001), with the same pattern of differences between the classes as for trait level scores.

The correlations of trait level with persons’ mean lnRT and AFQT scores also varied between classes. For example, the correlation between mean lnRT and trait level was moderately high in Class 1 and somewhat lower in Class 3. However, trait level was not significantly correlated with trait level in Class 2. For trait-level correlations with AFQT scores, Class 2 and Class 3 had similar moderate correlations, while Class 1 had a substantially lower correlation.

Table 3 presents descriptive statistics and regression results for the items within the three classes. As shown in Table 3, as typical in mixture models, the mean item difficulty is set to zero within each class. However, the standard deviation (SD) of item difficulties varied between classes. Class 1, with the lowest mean trait level, had the smallest item difficulty SD, while Class 2, with the highest mean trait level, had the largest item difficulty SD. Item response times showed similar patterns between classes. Class 1 had the lowest lnRT and smallest SD while Class 2 had the highest lnRT and largest SD. Thus, Class 1 has the least variability in item difficulty and response time while Class 2 has the most variability.

Table 3 also shows the differences between classes in the correlates of item difficulty. The correlation of item difficulty with lnRT was lowest for Class 1. Class 2 had the highest correlation and Class 3 had a somewhat lower correlation. The regression analysis for cognitive modeling shows a similar multiple correlation between classes as modeled from memory load and unique elements. Adding test position to the model increased the multiple correlation for all classes; however, the change was significant for Class 1 only. Further, notice that the beta value for the impact of memory load is somewhat smaller for Class 1 and unique elements had a borderline weight. Overall, the regression results for modeling item difficulty were very similar between Class 2 and Class 3.

Response time, on the other hand, had a different pattern of relationship between classes. First, response time was only marginally correlated with item difficulty in Class 1, while in the other two classes, it is a strong predictor (although somewhat lower in Class 3 than in Class 2). Second, the regression analysis for cognitive modeling of response time was not significant for Class 1; and adding test position did not increase the multiple correlation. However, Class 2 and Class 3 showed strong prediction in response times from the cognitive variables, with memory load being a significant and strong predictor. Also, adding the test position improved the response time prediction for both Class 2 and 3.

**Figure 2:**
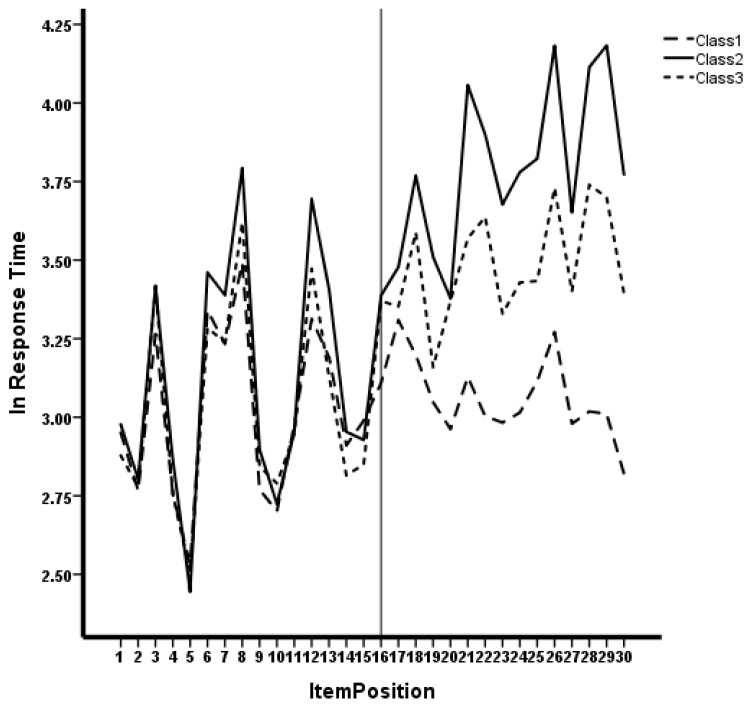
Item response time by position for three classes.

Figure 2 shows the relationship of lnRT with test position for the three classes. It can be seen that after the 16th item, Class 1 shows reduced variability and magnitude of lnRT compared to the other two classes. Also, Class 3 shows somewhat reduced variability and magnitude in lnRT, relative to Class 2.

To summarize, for all classes, item difficulty increased as cognitive complexity increased, especially as memory load increased. However, test position significantly increased the prediction of item difficulty from cognitive complexity variables only for Class 1. The prediction of item response time from the cognitive complexity variables varied substantially between classes. Response times were not significantly predicted from the cognitive variables for Class 1, and adding test position to the model did not significantly improve prediction. For Class 2 and 3, on the other hand, cognitive complexity showed substantial predictive value for the response time, and adding test position significantly increased this prediction.

### 2.4. Discussion

The latent classes differ in two aspects of validity: response processes and external relationships. Persons in Class 1 do not appear to be using an optimal strategy. Overall, the mean trait level is lower in this class relative to the other two classes, as is the mean item response time. Further, item difficulty is less related to item response time and the cognitive complexity variable of memory load than in the other classes. However, item difficulty is somewhat more highly related to test position for Class 1, indicating faltering performance toward the end of the test. Also, item response time is not significantly related to item memory load and test position for Class 1 as in the other two classes. Further, trait level in Class 1 is more highly related to mean item response time. Thus, examinees in Class 1 do not allocate the optimal amount of time for the more difficult items, particularly toward the end of the test. Finally, trait level is less correlated with the higher stakes external test (AFQT) than in the other two classes. These findings suggest that trait level has been lowered in Class 1 by not applying the optimal strategy for item solution throughout the test, but especially at the end of the test.

The other two latent classes were high-performing. Class 2 trait levels were associated with an extremely high probability of item solving (e.g., .844). Further, this class had the highest mean response time, with little variance between persons within the class. Class 3, although high-performing, had a lower mean trait level and mean response time. Further, persons varied more in mean response times within Class 3 than persons within Class 2, and response time was significantly correlated with trait level in Class 3. Also, persons in Class 3 had somewhat decreased response times toward the end of the test. The two classes had similar predictions of item difficulty and item response times from item cognitive complexity. Finally, for both classes, trait level had a moderate correlation with AFQT.

Overall, it appears that examinees in Class 1 needed some sort of modification in test procedures, monitoring, and/or instructions to ensure that their performance was more optimal throughout the test. Thus, the subsequent test use should consider altering the test administration conditions. For the current examinees, differential score interpretations by class are possible (i.e., performance level being optimal or not and differential performance correlations with external measures). Another possibility is to change the scoring method based on strategy shifts within the test. Study 2 explores this possibility.

## 3. Study 2: Intra-Individual Variability in Response Process

### 3.1. Background

As mentioned in the introduction, an approach to identifying within-person differences in shifts to construct-irrelevant responses is grounded upon the notion that both rapid and protracted response times are important indicators of alternative and noisy response processes. In contrast to the traditional notion of distinguishing strategy-driven response behavior from guessing behavior, the modeling in Study 2 uses a more general distinction between strategy-driven response behavior and noisy/erratic response behavior. While unexpectedly short response times may be indicative of disengaged, guessing behavior, unexpectedly long response times may be indicative of early processes in identifying and settling into an appropriate response strategy. For example, if an insufficient number of test items are presented for practice, some examinees may still be becoming familiar with the nature of the task and searching for the appropriate strategy when administering test items. Such a search procedure would be expected to produce exceedingly long response times for those items, as well as responses that are less informative to making claims about the construct of interest. Alternatively, as the duration of testing increases, fatigue and attentional lapses may have a greater influence over the response process, thereby producing extended response times and ability estimates which are less informative toward the construct of interest. The modeling approach in Study 2 can account for any number of transition processes, without making any strong a priori assumptions. Given these general principles, a formal presentation of our modeling approach is presented in the following sections.

Accuracy Model. As in Study 1, modeling accuracy data and deriving the latent ability estimates for the hypothetical construct, a Rasch modeling framework is used. The Rasch model adheres to the foundational measurement property of specific objectivity: person ability estimates may be estimated independent of the distribution of item difficulties, and therefore remain invariant across different item sets within the construct. For any given person and item, the probability of a correct response is a function of a simple additive relation between the person’s ability and the item’s difficulty:

(3) $$p(X_{ij}=1|\theta_{j},\beta_{i})=f\left(\theta_{j}-\beta_{i}\right)$$

where $p_{ji}$ represents the probability of a correct response on item i for person *j*; $\theta_{j}$ represents the latent ability of person *j*, and $\eta_{i}$ is the item difficulty of item *i*. For dichotomously scored items, responses are Bernoulli distributed and the logistic function is a common choice for *f* as shown in Equation (1).

However, if transitions occur between construct-driven and erratic response styles, then a measurement model that accounts for a mixture of response styles must be adopted:

(4) $$p(X_{ij}=1|\theta_{j},\beta_{i})=f\left(\theta_{j_{s}}-\beta_{i_{s}}\right)$$

Note that an additional subscript, *s*, has been affixed to the parameters of the model. This *s* subscript indicates that the different response styles will be represented by distinct parameter values. In this approach, *s* may take on two values (1, 2), corresponding to construct-driven and erratic response styles.

RT Model. As mentioned above, these different response styles are assumed to be strongly tied to different patterns in response times. In particular, *unexpectedly* long response times or *unexpectedly* short response times are both associated with a response style contaminated by the construction of irrelevant processes. However, even for principled, construct-driven responses, there are substantial individual differences in response time behavior. Furthermore, certain items induce longer response time behavior, depending on the item complexity and the associated processing demands. Thus, any designation of an ‘unexpected’ observed response time must be conditional on both person and item effects. Van der Linden (2006) proposed a response time model that accounts for both person and item effects, and we adopt his general approach as a foundation for deriving residual response time scores after conditioning on person and item effects. As in Study 1, log response time (lnRT) was used; thus, in the modeling of item *i* and person *j*

(5) $$\ln\left(RT_{ij}\right)\sim N\left(\mu_{ij},\sigma^{2}\right)$$

and the expected values of the transformed response times are an additive function of both person speed ($\tau_{j}$) and item time intensity ($\gamma_{i}$):

(6) $$\mu_{ij}=\tau_{j}-\gamma_{i}$$

Thus, $\sigma^{2}$ represents the residual variance in response times, after the person and item effects have been accounted for. In the case that both construct-driven and erratic response styles may be observed during a single testing occasion, one would anticipate some degree of heteroscedasticity to be observed. After an examinee makes the transition to an erratic response style, $\sigma^{2}$ would be expected to be quite large because the person has shifted to a response style inconsistent with their behavior during the other portions of the test. Thus, van der Linden’s model is extended to the following mixture case:

(7) $$\ln\left(RT_{ij}\right)\sim N\left(\tau_{j}-\gamma_{i},\sigma_{s}^{2}\right)$$

where the states (response styles) are defined by the size of the residual variance term (note the *s* subscript on $\sigma^{2}$ now). It is worth explicating here that although the accuracy and response time models are presented separately above, all parameters are estimated simultaneously in a joint model.

Modeling transitions. Given this framework, one can test different hypotheses regarding the pattern of transitions from one response style to another. For example, constraints can be applied such that transitions only occur at certain time points, or that only single transitions occur (e.g., single change-point model). Alternatively, if it is expected that multiple transitions occur during the course of a test (e.g., examinees transition into a construct-driven response style once task familiarity is sufficient, but then transition back into an erratic response style once time pressures emerge), then multiple change-point structure or Markov process might be integrated into the framework. A Markov process makes no assumptions regarding the number of change points, so it provides a useful exploratory technique for examining transition patterns. One simplifying assumption is made, however, in order to ensure a tractable solution: an examinee’s state (response style) for any given item, *i*, is dependent only on the state expressed for the previous item, *i* − 1, rather than the entire history of states. Put another way, a lag of only 1 is considered for the dependency structure of states expressed over time. Formally, if $\xi_{ji}$ represents the state expressed by subject *j* for item *i*, then

(8) $$P(\xi_{ji}=x_{ji}|\xi_{j1},\xi_{j2},\xi_{j3},\ldots ,\xi_{j,i-1})=P(\xi_{ji}=x_{ji}|\xi_{j,i-1})$$

For the empirical analysis, transitions are initially modeled according to such a Markov process. This allows an understanding of what response style examinees exhibit at the start of testing, whether transitions occur in a bidirectional or unidirectional manner, and the expected distribution of states if the test were to continue indefinitely. As will be discussed in more detail in the Section 3.4, a change-point process is also considered, based on the results of the Markov process analysis.

### 3.2. Method

**Test and examinees**. The same test and examinees as described in Study 1 above were used to examine the change-point model.

**Estimation.** All item and transition parameters were estimated via a fully Bayesian approach with MCMC methods and Gibbs sampling from the posterior with the JAGS: 4.3 software package. Normal priors were used for all item parameters, and a gamma prior was used for the response time residual variance terms. For initial state classifications at time 1 for subject *j* ($\xi_{j1}),$ values were drawn from a categorical distribution according to a pair of initial event probabilities:

(9) $$\xi_{j1}\sim cat\left(p_{1,state=1},p_{1,state=2}\right)$$

And the event probabilities (initial state probabilities) were set to a Dirichlet prior with concentration parameters fixed to 1:

(10) $$\left(p_{1,state=1},p_{1,state=2}\right)\sim Dirichlet\left(1,1\right)$$

And for state classifications at all remaining time points,

(11) $$\xi_{jt\neq 1}\sim cat(p_{t,state=1},p_{t,state=2}|p_{t-1,state=1},p_{t-1,state=2})$$

such that transitions follow a Markov process. These event probabilities (transition probabilities) are drawn again from a Dirichlet prior with concentration parameters fixed to 1:

(12) $$\left(p_{t,state=1},p_{t,state=2}|p_{t-1,state=1},p_{t-1,state=2}\right)\sim Dirichlet\left(1,1\right)$$

For the change-point analysis, a fully Bayesian approach with Gibbs sampling from the posterior was also implemented. Each subject’s vector of state classifications across items ($\xi_{j})$ is constrained to the following parameterization:

(13) $$\xi_{j}=\{\begin{array}{c} 1\;if\;i\leq k_{j}\\ 2\;if\;i>k_{j} \end{array}$$

where $k_{j}$ is an estimated parameter indicating the time point where a given examinee’s response style transition occurs. The $k_{j}$ values were then assumed to be drawn from a uniform prior distribution. In all cases, 10,000 samples were taken from the posterior after a period of 10,000 ‘burn-in’ iterations. Inspection of convergence plots, autocorrelation plots, and the Gelman-Rubin statistic ensured an appropriate burn-in and sampling interval.

Once all item parameters and state classifications were obtained, EAP estimates were derived for abilities, treating the item parameters and state classifications as fixed. Two ability estimates were obtained for each examinee; one for construct-driven states and one for erratic response states. Since only the ability estimates for construct-driven states were retained for further analysis, this effectively trimmed erratic responses from the data.

### 3.3. Alternative Trims

In order to evaluate the relative utility of emphasizing both positive and negative response time residuals in identifying threats to validity, we consider a few additional trims based on alternative criteria. First, we consider a test trim based on only negative residuals. If simple rapid guessing processes can fully account for any threats to validity, then eliminating only highly negative residuals should produce ability estimates with improved relationships using external measures. To explore this possibility, we evaluate the correlation between ability estimates and the AFQT scores across several different trims based on the magnitude of negative residuals (<−6 SD, <−5 SD, <−3.5 SD, <−3 SD, <−2.5 SD, <−2 SD, <−1.5 SD, <−1 SD, <−.5 SD, and < 0 SD).

Second, in order to rule out the possibility that disengaged responding may be captured by individual mean shifts in response time over the course of a test (rather than a shift towards erratic responding in either direction), we consider a change-point model based on individual shifts in the person speed parameter, $\tau_{j}$:

(14) $$ln\left(RT_{ij}\right)\sim N\left(\tau_{j_{s}}-\gamma_{i},\sigma^{2}\right)$$

which is similar to our proposed model in Equation (7), except with the states defined with respect to the person speediness parameter rather than the residual parameter. This change-point model is similar to what Zhu et al. proposed in (Zhu et al. 2023).

### 3.4. Results

Can the transition patterns be directly modeled and hence used to inform future test administration practice? An initial analysis using a Markov process transition model indicated that the vast majority of examinees began the test with a construct-driven response style, and then transitioned towards a more erratic response style (see Table 4 for the estimated initial and stationary distribution of response style states). At the onset of testing, nearly 95% of examinees were responding to ART items in a seemingly consistent and principled fashion (log-transformed residual variance, $\sigma_{1}^{2}$ = .206). However, if continued to test indefinitely, then this proportion is expected to drop to 70%.

**Figure 3:**
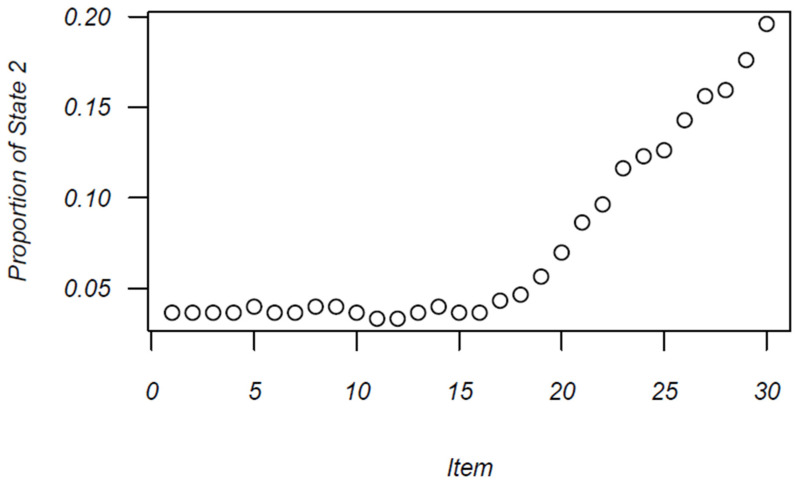
Proportion of examinees in erratic state across items.

Figure 3 displays the increase in the proportion of examinees engaged in erratic response styles over the course of the testing window. Figure 2 shows somewhat of an elbow; the proportion of erratic response styles remains low and unchanged for the first half of the test, and then begins to precipitously increase after item 16, after which there is a sharp rise in erratic responding. Further, 20% of individuals have transitioned to the erratic response state by the final item in the test.

Together, these results support a potential change-point process, such that virtually all examinees begin responding to items in a seemingly consistent and principled way, and then transition into more erratic response styles as the test proceeds (perhaps as fatigue, attentional lapses, and guessing behavior influence the response process). Therefore, we re-examine the pattern of response style states and transitions using a change-point model.

**Figure 4:**
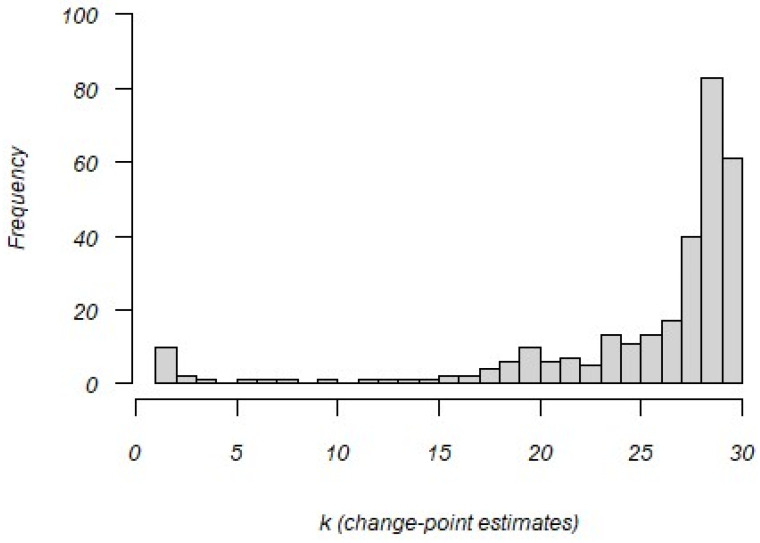
Distribution of change-point (k) estimates for 301 examinees.

Figure 4 shows the distribution of the estimated change points based on changes in log-transformed residual response time variances. Note that these indicate change points in transitioning from a construct-driven response style to an erratic response style. The vast majority of change points occur late in the testing window, indicating testing fatigue may play a prominent role in affecting the response process.

**Figure 5:**
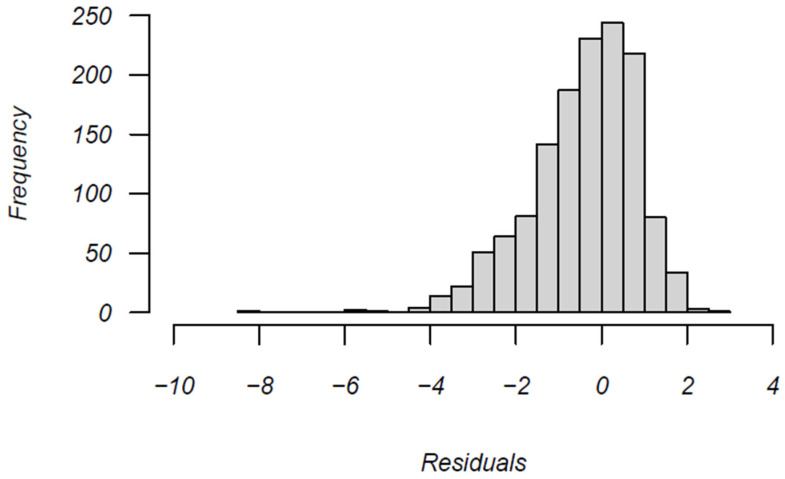
Distribution of residuals after change point (k) for 301 examinees.

Table 5 shows that after the transition, the magnitude of the log-transformed residual variance increases by a factor of almost 9, suggesting that towards the end of the test, examinees demonstrate dramatically inconsistent patterns of responding. Furthermore, the probability of a correct response post-change-point decreases to .26 from the pre-change-point value of .65, further underscoring the notion that the pattern of transition is from optimal to non-optimal. Note that Figure 5 shows the distribution of residual values after the change point. While the distribution is negatively skewed (indicating the presence of some extremely unexpected short response times; perhaps characteristic of rapid guessing behavior), there also exists a substantial number of positive values, indicating that a singular mean shift in response speed is unable to account for the unexpected response times.

Can information from these transition patterns be used to adjust the ability estimates in an effort to improve the underlying validity of the measure? Having established the pattern and direction of these transitions, the next logical question is whether or not this information can be used to isolate the construct-driven responses and improve the validity coefficient of the test. The second column of Table 6 displays the correlation coefficient between the corrected ability estimates (i.e., after erratic responses have been trimmed according to the change-point model) and the AFQT general score. Note that the table displays the correlation coefficient after it has been adjusted to account for the degree of unreliability in the ART test post-trim (see Appendix A for more information). The corrected ability estimates account for almost 40% of the variance in AFQT general scores, a full 8% gain in variance accounted for over and above ability estimates derived from the non-trimmed ART test. Additionally, this gain was statistically significant at *p* < .05 (see Appendix A for a description of the bootstrapping method used to construct the associated null distribution for the significance test). Also, note that the corrected ability estimates here demonstrate an improved relationship with the AFQT scores, relative to the cluster ability estimates from Study 1 (see Table 2).

**Figure 6:**
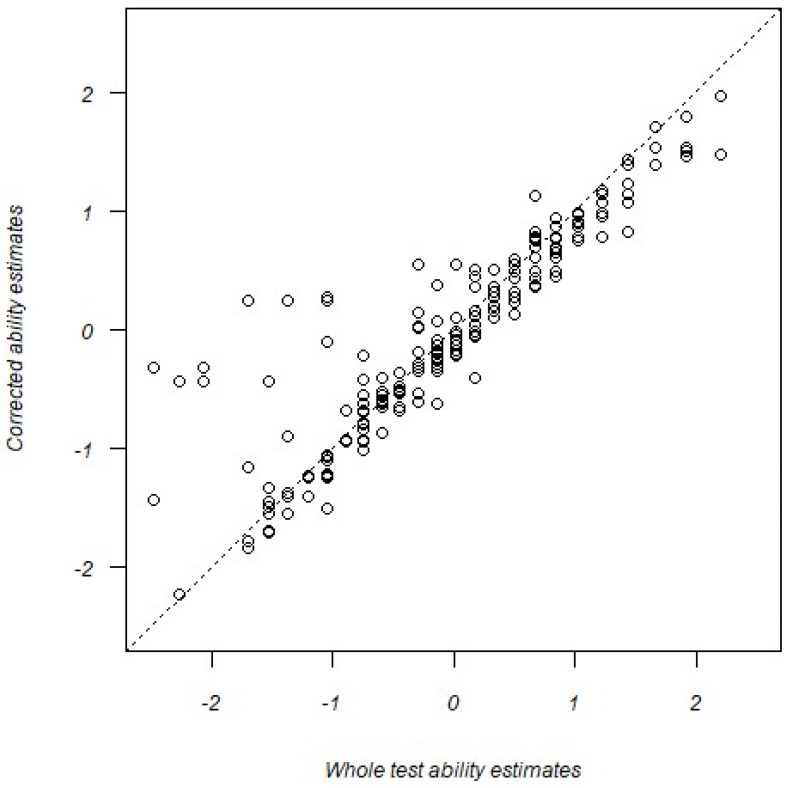
Relationship between corrected ability estimates and whole test ability estimates.

The gain in the predictive quality of the trimmed ART test seems to be a function of underestimated abilities in the original whole test analysis. Figure 6 shows a scatterplot of corrected ability estimates from the trimmed data against the traditional, whole test ability estimates. Note that although most points fall along the diagonal (indicating invariance in the ability estimates), a sizable number of points fall *above* the diagonal, particularly at the lower end of the original (whole test) ability distribution. By excluding potentially erratic responses, a subset of ability estimates were boosted upward, producing stronger correlations with the AFQT score.

A critical question is whether the recommended use of response time residual variance as an index of construct-irrelevant responding provides any utility over an approach that simply focuses on guessing and rapid responding behavior. To evaluate whether protracted response times are additionally informative towards identifying disengaged responding, ART data were trimmed according to only *highly negative* residual response times, and the correlation between the resulting ability estimates and AFQT general scores was evaluated. These results are displayed in Table 6. Regardless of the threshold used to define an uncharacteristically short response time, none of the resulting trims produced significant improvements in the validity coefficient. Furthermore, a test trim informed by a change-point model based simply on individual-level mean shifts in response speed (similar to what (Zhu et al. 2023) proposed) produced a smaller improvement in the validity coefficient than our trim based on the residual analysis. Only when data trims were informed by *both* uncharacteristically small and uncharacteristically long response times was there a significant improvement in the validity coefficient.

### 3.5. Discussion

The approach outlined above adopted contemporary concepts from the psychometric modeling of response times and formalized these concepts into a time series measurement model to derive corrected ability estimates with clearer interpretive value. In particular, large residual response times, after conditioning on person and item effects, were used as a potential indicator of erratic response styles. Van der Linden and Guo (2008) had previously suggested the use of similar residual terms as a potential indicator of aberrant responding, and we extended the notion into a formal approach that (1) identifies how erratic responding tendencies may unfold over time and (2) produces ability estimates that are less contaminated by erratic response processes.

With respect to ability estimates with clearer interpretive value, an empirical analysis with the analogical reasoning items demonstrated an improved validity coefficient once erratic responses were removed from the analysis. Note that these corrected ability estimates also demonstrated an improved validity coefficient over the cluster-specific ability estimates derived from Study 1. Furthermore, nearly all examinees began the testing process with a construct-driven response style, with a net gain in erratic responses over time, implying a change-point process. This case suggests that administering additional items may not be a useful approach to improving the psychometric qualities of the test, as response processes were already compromised by the current length of the test.

From a broader perspective, the results speak to the importance of implementing such a procedure in practical testing settings to guide future administrations and obtain the construct-pure estimates of ability. It also underscores the importance of considering both uncharacteristically short *and* uncharacteristically long response times as evidence of erratic responding. Simply emphasizing guessing and rapid responding styles (as is often the case in explorations of response styles; see (Molenaar and DeBoeck 2018; Qian et al. 2016; Wise and DeMars 2006) for a few examples) has the potential to neglect other types of disengaged responding, producing biased estimates of ability. Our method, in contrast, additionally considers uncharacteristically long response times as evidence of construct-irrelevant influences on the response process, and the utility of this approach was established empirically.

One additional note is worth mentioning regarding the interpretation of the engaged versus erratic states. If two states are identified with sizable differences in the response time residual variance, one might immediately assume that the state with the larger residual term is reflective of an erratic or non-optimal response process. However, this may not necessarily be the case, and the inspection of additional model parameters is required in order to develop a more valid characterization of the states. An observation with a large residual variance term simply indicates that the recorded response time was quite unexpected, given a subject’s general pattern of responding on all other items on the test. For example, if a subject engages items with a principled solution strategy for most items, but becomes fatigued towards the end of the test and responds with rapid guessing behavior for the last few items, then these last few observed response times would produce large residuals. In this case, it is clear that the state with a large residual variance term is reflective of a disengaged response style. However, it is alternatively possible that an examinee may be engaged for only the first few items, and then begin rapidly guessing for the vast majority of items that follow. In this latter case, the longer response times for the first few items represent the exception, and would actually produce the larger residual values. Thus, there is the potential for a state with increased residuals to actually reflect a more engaged, principled response process. In order to disentangle the two possibilities, it is helpful to examine the parameters from the accuracy model. A disengaged response style should produce much higher rates and increased item difficulties; so, these parameters can be evaluated to confirm the suspected nature of the two response states. In our application, the error rates and item difficulties increased precipitously after the change point when inflated residual values were observed, implying a general transition towards disengaged responding for the last remaining items.

## 4. Discussion and Summary

Both Study 1 and Study 2 evaluated potential threats to validity when traditional unidimensional psychometric models are applied to real test data from non-verbal ability tests on analytic reasoning. However, each study evaluated these threats through a different lens. Study 1 employed mixture IRT modeling, which seeks to identify between-person classes that reflect heterogeneity in response patterns. Study 2, conversely, employed recent developments in response time modeling which examine response and response time vectors as time series data with potential *intra-individual variability* in response style.

Study 1 was able to identify between-person heterogeneity in response patterns, implying fundamentally different response processes across classes which obfuscated the meaning of performance estimates (and impacted the relationship between performance estimates and external measures). Class 1, for example, exhibited fast response times which had a diminished correlation with item complexity factors. Also, item difficulty was not as strongly modeled by cognitive complexity. Furthermore, Class 1 showed a marked decrement in performance, relative to the other classes. These findings might imply that response behavior in Class 1 is often governed by a less strategy-oriented process (e.g., fatigue and guessing), a claim that is further supported by the precipitous decline in response times for later items in the test. In this case, the potential for ability estimates from Class 1 to predict performance in other contexts would be compromised. In fact, the correlation between the ability estimates and performance on a high-stakes test of mathematical and verbal skills was quite weak for this class. Practically, the results from such an analysis would suggest that ability estimates from the different classes should not follow a unitary interpretation, and perhaps additional testing procedures should be considered. For example, both instructions and test monitoring procedures might be amplified to help ensure that construct-relevant processing is employed throughout the test.

However, in order to provide testing recommendations and modifications tailored at the *individual* level, additional psychometric modeling approaches are required that can reliably identify both inter- and intra-individual variability in response processes. Study 2 proposed such a psychometric approach by directly leveraging response time information and a time series perspective. While Study 1 was able to provide emerging evidence that there may be some variability in the unfolding of non-optimal or disengaged response styles over the course of the test, Study 2 clarified these trends at the individual level by increasing the degree of resolution with which these processes were identified and evaluated. In particular, Study 2 evaluated within-person changes in engaged versus erratic responding using an analysis of response time residuals and simultaneously captured how the pattern of these changes differed across people. Furthermore, the higher resolution approach of Study 2 was able to produce corrected ability estimates, which showed statistically reliable improvements in the relationship with performance estimates from a high-stakes test of mathematical and verbal skills. Critically, this work proposed a useful time series psychometric approach to validity evaluations and contrasted this with classic, mixture modeling approaches that may have insufficient resolution to provide individualized recommendations towards improving validity. It also highlighted the utility of a response time residual-based approach in identifying how threats to validity unfold over time at the individual level.

By providing a more refined, detailed account of individual deviations from a singular response process, researchers, test developers, and test administrators are in a better position to understand how construct-irrelevant processes may affect response behavior, and make adjustments accordingly. In some contexts, this may develop a test trim, similar to what was achieved in Study 2, in order to obtain ability estimates that are more pure representations of the latent trait of interest. Alternatively, if it is undesirable to adapt the instrument to the test taker’s behavior, then this psychometric approach can still provide valuable information regarding optimal test length and how examinees may improve performance in subsequent administrations.

## Figures and Tables

**Table 1:** Mixture model statistics for ART.

Classes	Class Sizes	-2lnL	Number Parameters	AIC	Χ^2^
One class	1.00	9664.96	31	9727.69	---
Two class	.61, .39	9564.86	63	9690.85	100.10 **
Three class	.46, .31, .23	9506.48	95	9696.49	58.38 *

Note: ** *p* < .01, * *p* < .05.

**Table 2:** Descriptive statistics and correlations for persons within classes.

Class Variable		Mean	SD	Correlations MnlnRT AFQT	
1 N = 135	Trait	−.235	.889	.656 **	.231 *
	Mn lnRT	3.038	.611	1.000	.143
	AFQT	214.510	15.440	.143	1.000
2 N = 98	Trait	1.689	.819	.109	.511 **
	Mn lnRT	3.446	.289	1.000	.119
	AFQT	230.121	15.485	.119	1.000
3 N = 68	Trait	.746	.866	.515 **	.509 **
	Mn lnRT	3.251	.362	1.000	.239
	AFQT	223.064	16.880	.239	1.000

Note: * *p* < .05, ** *p* < .01.

**Table 3:** Descriptive statistics and correlations for items within classes.

				Regression: Cognitive Modeling				
Variable Class		Mean	SD	r_lnRT_	R	Memory Load β1	Unique Elements β2	Position Added R
Item Difficulty	1	.000	1.201	.444 *	.613 **	.469 **	.302 ^+^	.717 **
	2	.000	1.890	.754 **	.607 **	.534 **	.190	.645 **
	3	.000	1.548	.642 **	.613 **	.512 **	.240	.629 **
Response Time	1	3.038	.215	1.000	.128	.038	.114	.130
	2	3.445	.470	1.000	.659 **	.607 **	.153	.765 **
	3	3.250	.342	1.000	.631 **	.583 **	.142	.697 *

Note: ^+^ *p* < .10, * *p* < .05, ** *p* < .01.

**Table 4:** Initial and stationary distributions for construct-driven and erratic States.

	Initial State Distribution	Stationary Distribution
State 1: Construct-driven	.948	.700
State 2: Erratic response	.052	.300

**Table 5:** State-specific RT residual variance and accuracy estimates.

State	$\sigma^{2}$	$p\left(X_{ij}=1\right)$
Construct-driven	0.187	.67
Erratic	1.647	.26

Note. $\sigma^{2}$ represents the EAP estimate from the posterior.

**Table 6:** Correlations and variance accounted for in AFQT scores by ART ability estimates from various data trims.

		*r*	*% Variance*
Original Data (No Trim)		.559	.312
Change-point Model (residual analysis)		.628 *	.394
Change-point Model (response speed shift)		.566 *	.320
Markov Process Model		.591 *	.349
Data with removal for:			
	All negative residuals	.565 *	.319
	<−0.5 SD residuals	.550 *	.303
	<−1.0 SD residuals	.569 *	.324
	<−1.5 SD residuals	.565 *	.319
	<−2.0 SD residuals	.568 *	.317
	<−2.5 SD residuals	.563 *	.319
	<−3.0 SD residuals	.563	.317
	<−3.5 SD residuals	.562	.316
	<−5.0 SD residuals	.559	.312
	<−6.0 SD residuals	.559	.312

* *p* < .05.

## Data Availability

The original contributions presented in the study are included in the article, further inquiries can be directed to the corresponding author.

## Appendix A. Reliability Adjusted Validity Coefficient

The ultimate purpose of distinguishing between construct-driven response styles and erratic response styles is to derive ‘uncontaminated’ estimates of ability; that is, ability estimates that are not confounded by construct-irrelevant processes. Eliminating these items should theoretically strengthen the observed validity coefficients. However, trimming items may severely restrict the reliability of the test, thereby attenuating the validity coefficient. In order to properly evaluate the validity of an instrument after a trim is made, the observed validity coefficient must be corrected to account for the loss in reliability produced by the trim.

### Appendix A.1. Calculation

Within the realm of classical test theory, the basis for such an adjustment is relatively straightforward: the adjusted validity coefficient is simply the raw, observed validity coefficient divided by the root of the product of the two reliability coefficients for the instruments being compared:

$$\rho_{xy}^{*}=\frac{\rho_{xy}}{\sqrt{\rho_{xx}*\rho_{yy}}}$$

where $\rho_{xy}^{*}$ is the adjusted validity coefficient, $\rho_{xy}$ is the observed validity coefficient (calculated correlation between a measure and some criterion the measure is theoretically associated with), and $\rho_{xx}$ and $\rho_{yy}$ are the reliability coefficients for the measure and criterion, respectively. Additionally, note that the reliabilities are a function of the standard errors of the instrument:

$$\rho_{xx}=\frac{\sigma_{\theta }^{2}-SE_{\theta }^{2}}{\sigma_{\theta }^{2}}$$

where $\sigma_{\theta }^{2}$ is the observed variance of the ability estimates, and $SE_{\theta }^{2}$ is the squared standard error of the measure. However, note that adjusting the validity coefficient is not as straightforward when trims are made as detailed above. Each examinee might show different patterns of transitions between construct-driven and erratic response styles, producing different trimming patterns, and therefore different standard errors for each subject’s response vector. In order to derive a broad measure of the standard error of the instrument after individualized trims, we first calculate the squared standard error of each examinee’s ability to estimate post-trim, and then take the mean of the squared standard errors. The equation above is then adjusted correspondingly:

$$\rho_{xx}=\frac{\sigma_{\theta }^{2}-\bar{SE_{\theta }^{2}}}{\sigma_{\theta }^{2}}$$

### Appendix A.2. Testing for Significance

In order to determine whether or not the trim informed by the model actually produces a statistically significant improvement in the validity coefficient, we need to consider what the expected adjusted validity coefficient is under the null condition. In this analysis, the null condition corresponds to the case when a trim is unrelated to the response style and is completely randomized. If the model-informed trim is performing no better than a randomized trim, then its utility cannot be justified.

To develop a distribution of null results, we use a bootstrapping method to produce a set of adjusted validity coefficient values from randomized trims. In particular, once an optimal trim is identified based on residual response times, we then randomize the assignment of these trims across persons and items to derive a new (null) adjusted validity coefficient. The trims are randomized again to produce another (null) adjusted validity coefficient. This is performed iteratively until a full distribution of null adjusted validity coefficients is constructed and then used as a basis for evaluating the magnitude of the adjusted validity coefficient from the model-informed trim.
